# Evaluating the Diagnostic Test Accuracy of Molecular Xenomonitoring Methods for Characterizing Community Burden of Lymphatic Filariasis

**DOI:** 10.1093/cid/ciab197

**Published:** 2021-06-14

**Authors:** Joseph Pryce, Lisa J Reimer

**Affiliations:** Department of Vector Biology, Liverpool School of Tropical Medicine, Liverpool, United Kingdom

**Keywords:** xenomonitoring, lymphatic filariasis, mosquito

## Abstract

**Background:**

Molecular xenomonitoring (MX), the detection of pathogen DNA in mosquitoes, is a recommended approach to support lymphatic filariasis (LF) elimination efforts. Potential roles of MX include detecting presence of LF in communities and quantifying progress towards elimination of the disease. However, the relationship between MX results and human prevalence is poorly understood.

**Methods:**

We conducted a systematic review and meta-analysis from all previously conducted studies that reported the prevalence of filarial DNA in wild-caught mosquitoes (MX rate) and the corresponding prevalence of microfilaria (mf) in humans. We calculated a pooled estimate of MX sensitivity for detecting positive communities at a range of mf prevalence values and mosquito sample sizes. We conducted a linear regression to evaluate the relationship between mf prevalence and MX rate.

**Results:**

We identified 24 studies comprising 144 study communities. MX had an overall sensitivity of 98.3% (95% confidence interval, 41.5–99.9%) and identified 28 positive communities that were negative in the mf survey. Low sensitivity in some studies was attributed to small mosquito sample sizes (<1000) and very low mf prevalence (<0.25%). Human mf prevalence and mass drug administration status accounted for approximately half of the variation in MX rate (*R*^2^ = 0.49, *P* < .001). Data from longitudinal studies showed that, within a given study area, there is a strong linear relationship between MX rate and mf prevalence (*R*^2^ = 0.78, *P* < .001).

**Conclusions:**

MX shows clear potential as tool for detecting communities where LF is present and as a predictor of human mf prevalence.

There are 65 million people currently infected with lymphatic filariasis (LF) worldwide [1, 2], making it the second-most-common vector-borne disease after malaria. The disease is associated with inflammation and dysfunction of the lymphatic system leading to severe pain and the development of chronic symptoms. More than 90% of LF cases are caused by the filarial nematode *Wuchereria bancrofti*, which is prevalent in many tropical and subtropical areas. Species from 3 major mosquito genera can act as vectors for *W. bancrofti*: *Culex pipiens quinquefasciatus* in urban areas, *Anopheles* species in rural areas of Africa, and *Aedes* species in the South Pacific [3].

The Global Program to Eliminate Lymphatic Filariasis (GPELF) was launched in 2000 to eliminate LF as a public health problem through mass drug administration (MDA) of preventative chemotherapy and morbidity management to alleviate suffering [4]. The drugs used in MDA do not kill the adult worms and instead target the juvenile microfilariae (mf) that are transmissible to mosquitoes. It is therefore necessary to repeat MDA for a minimum of 5 years, the duration of the adult worm lifespan, in order to interrupt transmission. Despite significant progress, LF has been eliminated from just 16 of the 72 previously endemic countries or territories, while a further 7 countries have completed the required number of MDA campaigns [5]. The target for elimination as a public health problem was recently updated from 2020 to 2030 [6].

## Lymphatic Filariasis Surveillance

Traditional LF surveillance involves screening the human population for the presence of mf, LF antigens, or host antibodies [7]. If, after completing a program of MDA, an implementation unit records either an mf prevalence of less than 1% or antigen prevalence of less than 2% at each sentinel and spot check site, a transmission assessment survey (TAS) is conducted. The TAS determines whether the antigen prevalence is less than 2% in 6- to 7-year-old children, an indicator that transmission has been successfully interrupted and MDA can be stopped. The TAS is repeated during post-MDA surveillance to ensure the interruption of transmission has been sustained [7].

These tools are not without their limitations. Antigen or antibody tests are unable to differentiate between current or prior infections. Microfilaria detection through microscopy or polymerase chain reaction (PCR) captures current infections but must often be conducted at night due to the nocturnal periodicity of many filarial strains. Furthermore, their sensitivity for detecting areas with very low but persistent transmission has been brought into question by a number of examples in which achieving the 1% target did not lead to elimination and prevalence rebounded [8–11]. In addition, obtaining human biological samples is invasive, resource intensive, and operationally challenging at large scales [12]. Such costs may be justified when the prevalence of a disease is high, but as progress towards elimination is made and the number of cases identified per survey decreases, funding bodies and at-risk populations may lose enthusiasm for the continued use of invasive and expensive methods.

## Xenomonitoring

While entomological surveillance for vector-borne diseases typically involves the detection of infected or infective vectors to assess disease transmission, molecular xenomonitoring (MX) involves the detection of pathogen DNA in the vector and is a proxy for the presence of the pathogen in the human population [13]. The use of MX as a surveillance tool overcomes many of the challenges associated with case surveillance as it does not rely on human blood sampling, it is relatively inexpensive, is indicative of current infections, and is amenable to integrated surveillance of multiple diseases [14].

The World Health Organization recommends MX to be incorporated into LF surveillance strategies and it is increasingly being used to lend support to program decisions [15]. A recent meta-analysis showed that over 300 000 mosquitoes have been collected and analyzed for infection [16]. However, there is no standardized approach for conducting MX surveillance.

Developing a systematic strategy for MX first requires clarification of its intended use(s). While MX methods cannot be used to identify whether individual humans are positive for LF, there are 2 distinct ways in which MX can support the surveillance activities of elimination programs. First, MX may be used to determine whether LF is present in communities, particularly in areas of very low prevalence where cases may not be detected by TAS. Second, it may serve as a proxy for human prevalence and help monitor progress towards elimination. However, the sensitivity of MX to detect LF-positive communities, in comparison to traditional human sampling methods, has never been evaluated. In addition, the relationship between MX rates (defined as the proportion of the mosquito population that is positive for LF DNA) and human prevalence is poorly understood. Program decisions to stop or re-instate MDA continue to be based on specific estimates of human infection prevalence, which MX surveys are currently unable to provide.

The primary aim of this systematic review and meta-analysis is to assess the sensitivity of MX to detect areas of above zero LF prevalence and explore the factors that affect sensitivity. A secondary aim was to evaluate the relationship between mf prevalence and MX rates in areas of above zero prevalence and determine whether MX rates reflect changes in human mf prevalence.

## METHODS

For this review and meta-analysis, we followed the Preferred Reporting Items for Systematic Reviews and Meta-Analyses (PRISMA) guidelines [17]. The review follows a protocol registered with the PROSPERO international database of prospectively registered systematic reviews in health and social care (CRD42020200351).

### Search Strategy

We searched 5 online bibliographic databases incorporated into EBSCOhost (CINAHL Complete, MEDLINE Complete, Global Health, eBook Collection, Global Health Archive) for records published up to 9 July 2020. The search strategy is presented in Supplementary Table 1. We additionally searched the reference lists of all identified articles.

### Inclusion Criteria

Primary research studies were suitable for inclusion if they (1) collected wild mosquitoes of any genus and used molecular methods to report the MX rate and (2) reported the mf prevalence in the human population living in the area where mosquitoes were collected. We excluded studies where measurements of MX rate and mf prevalence were taken more than 18 months apart or if MDA was distributed in the study area between the 2 time points.

### Data Extraction and Management

After initial screening of the titles and abstracts of identified articles, the full texts of potentially relevant studies were read and evaluated against the inclusion criteria. Information from the included studies was extracted using prepared proformas. Each stage was completed by J. P., with areas of uncertainty discussed with L. J. R.

We extracted data on the study setting, objectives, history of MDA and other interventions, details of the primary vector and parasite species, and methods used for sampling and screening mosquito and human populations. In case of missing data, we attempted to contact study author(s) for clarification.

Where studies reported outcome data at subunits of the overall geographical area covered (eg, individual villages within a district), we extracted information at the smallest available level. For each study area, we recorded the mf prevalence, MX rate, and binary measures of the presence or absence of filaria-positive mosquitoes and humans. Where necessary, we estimated the MX rate from the reported data using PoolScreen v2.0 [18]. Where studies screened different mosquito genera separately, we combined the survey results to determine the presence or absence of positive mosquitoes and overall MX rate.

We assessed each study’s methodological quality for answering the review objectives using a checklist adapted from the QUADAS-2 tool [19]. Studies were evaluated based on 5 assessment criteria: whether those interpreting mf survey results were blinded to the MX results and vice versa, the length of time between surveys, the degree to which the 2 sampling strategies targeted the same communities, and the continuity of methodology between sampling time points (longitudinal studies only). For each criterion, studies were graded as high, low, or unclear risk of bias based on predetermined specifications (Supplementary Table 2). The assessments were conducted by J. P., with areas of uncertainty discussed with L. J. R.

### Statistical Analyses

To evaluate the sensitivity of MX, we treated study areas as the unit of observation and used typical diagnostic test evaluation methods. We considered MX results as the index test and mf survey results as the reference standard to calculate the number of true positives, true negatives, false positives, and false negatives in each study. We used a bivariate model utilizing the *metandi* and *xtmelogit* commands in Stata version 14 (StataCorp) to obtain a pooled estimate of the sensitivity of MX. We compared the MX sensitivity between surveys of varying mosquito sample sizes and at a range of mf prevalence values. Confidence intervals (CIs) were calculated using the Wilson method [20]. We did not evaluate MX specificity due to known limitations in the sensitivity of the reference standard and strong evidence that molecular detection methods are highly specific [21]. We instead report the number of areas in which positive mosquitoes were detected despite mf surveys reporting zero positive humans.

To evaluate the relationship between mf prevalence and MX rate, we conducted a linear regression. We included covariates for primary vector genus and progress towards elimination and weighted the regression by mosquito sample size. To determine whether MX rates reflect changes in mf prevalence within a given study area, we conducted a further linear regression incorporating data from longitudinal studies only and including covariates for sampling time point and study site. Models were constructed using the lm() function in R version 3.6.2.

## RESULTS

### Search Results

**Figure 1.**
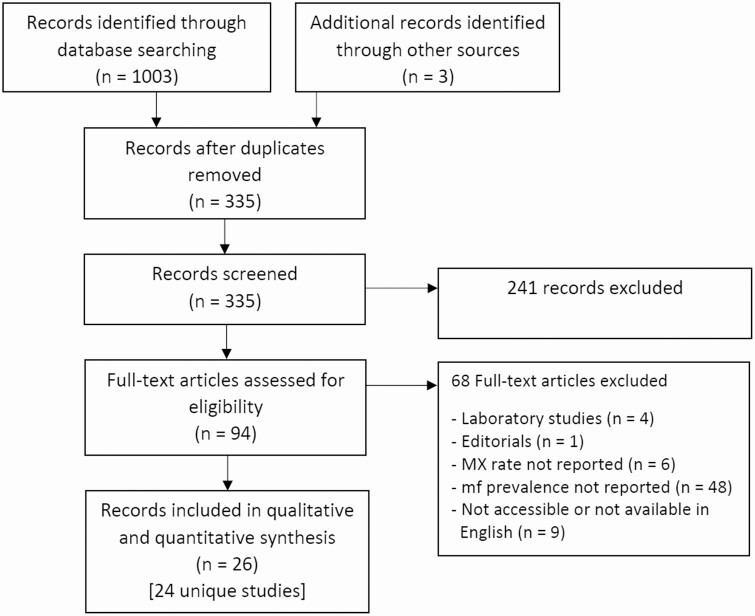
Results of the search and reasons for exclusion of studies. Abbreviations: mf, microfilaria; MX, molecular xenomonitoring.

The electronic search strategy identified a total of 1003 records. A further 3 records were identified by other sources. After removal of duplicates, 335 records were screened. A total of 26 records corresponding to 24 unique studies met the inclusion criteria for the review (Figure 1) [8, 9, 22–45].

### Details of Included Studies

**Figure 2.**
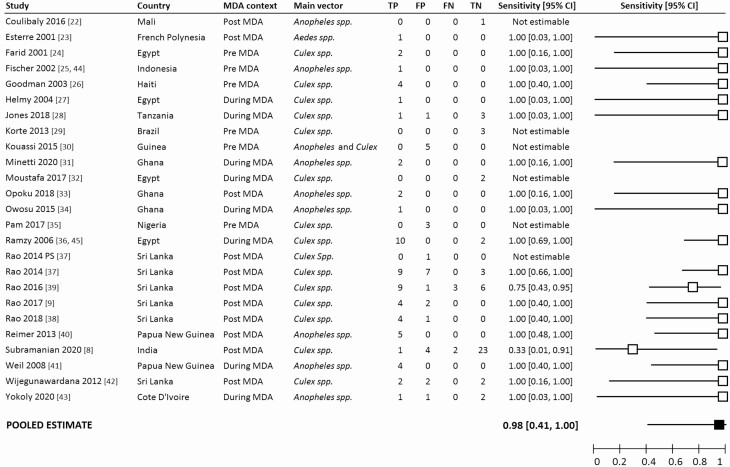
Forest plot summarizing the study details and the sensitivity of MX for detecting communities that were positive for LF as determined by microfilaria surveys. The pooled estimate of sensitivity is indicated by the black square. Abbreviations: CI, confidence interval; FN, false negatives; FP, false positives; MDA, mass drug administration; PS, preliminary survey; TN, true negatives; TP, true positives.

Included studies had been conducted across a variety of geographical settings, primary vector species, and elimination stages (Figure 2).

The objectives of the included studies were wide-ranging. The most common aim was to identify the presence or map the distribution of LF (12 studies). Six studies aimed to evaluate the usefulness of MX methods or compare MX results with other surveillance methods. Other objectives included measuring the impact of MDA implementation (3 studies) or insecticide-treated net distribution (1 study) on LF indicators, and evaluating the field use of novel molecular detection methods (4 studies) or trap types (1 study).

All studies screened mosquito carcasses for filarial DNA. One study additionally reported the prevalence in mosquito excreta/feces [31]. However, for consistency with the other studies, we only included the mosquito carcass MX rate in our analyses. The methods used for trapping mosquitoes, pooling, and DNA extraction and amplification varied greatly between studies. A full description of these methods is presented in Supplementary Dataset 1.

Across the 24 included studies, MX and mf survey data were available for 144 distinct areas, ranging in size from the district to village level. The median number of people surveyed in each area was 509.5 (range, 41 to 3795). The median number of mosquitoes surveyed was 1258 (range, 23 to 5280). Three studies and 4 study sites provided longitudinal data with a minimum of 3 sampling time points [36, 37, 41].

### Assessment of Methodological Quality

Overall, there were few concerns about methodological quality across the included studies. In most studies, the mf and MX surveys were conducted within 6 months of one another, although 2 studies conducted the surveys approximately 12 to 18 months apart [8, 39]. In 2 studies, the MX and mf surveys did not specifically target the same communities within the study site [30, 39]. There were no concerns about the continuity of methodology in longitudinal surveys. Five studies limited their mf surveys to specific populations and the estimate of prevalence may therefore have limited applicability to the wider population. The quality assessments for each included study are provided in Supplementary Figure 1 and Supplementary Table 3.

### Evaluating the Sensitivity of Molecular Xenomonitoring Methods

Positive mosquitoes were identified in 92 of the 144 surveyed areas (63.9%). The overall sensitivity of MX for detecting mf-positive areas was 98.3% (95% CI, 41.5–99.9%) (Figure 2). In addition, MX detected positive mosquitoes in 28 areas where mf surveys failed to detect any positive individuals.

**Figure 3.**
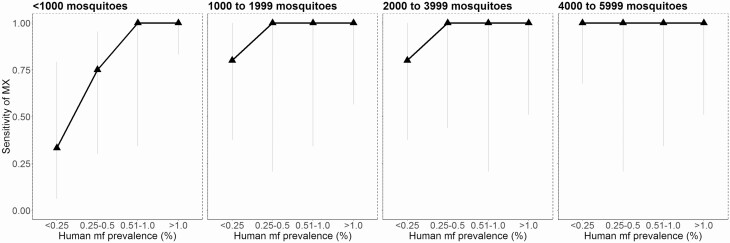
MX sensitivity for detecting communities that are positive for LF (as determined by mf surveys) at a range of mf prevalence and with varying mosquito sample size. Abbreviations: LF, lymphatic filariasis; mf, microfilaria; MX, molecular xenomonitoring.

The sensitivity of MX at a range of human mf prevalence values and mosquito sample sizes is shown in Figure 3. Where the human mf prevalence was very low (<0.25%), MX surveys screening fewer than 1000 mosquitoes had a sensitivity of .33 (95% CI, .06–.79). However, sensitivity increased to .80 (95% CI, .38–1.00) when screening either 1000–1999 or 2000–3999 mosquitoes, and to 1.00 (95% CI, .67–1.00) when screening 4000–5999 mosquitoes. At low mf prevalence levels (>0.25% to 0.5%), surveys of fewer than 1000 mosquitoes had a sensitivity of .67 (95% CI, .30–.95), but all surveys screening more than 1000 mosquitoes had a sensitivity of 1.00. Where the human mf prevalence was moderate (0.51–1%) or high (>1%), MX sensitivity was 1.00, regardless of the number of mosquitoes screened.

### Correlation Between Molecular Xenomonitoring Rate and Microfilaria Prevalence

**Figure 4.**
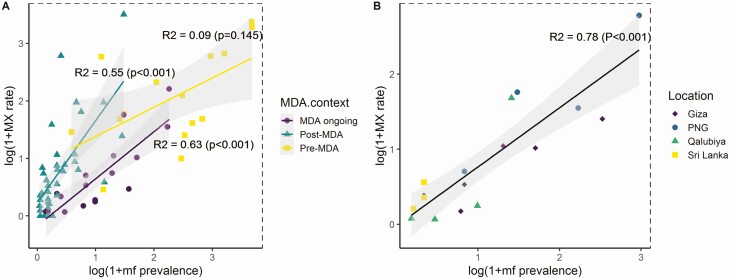
*A*, Linear regression models demonstrating the relationship between human mf prevalence and MX rate in a variety of elimination phases. *B*, Linear regression model demonstrating the relationship between human mf prevalence and MX rate when consistent methods are used for sampling and screening of humans and mosquitoes (using data provided by longitudinal studies only). Abbreviations: mf, microfilaria; MX, molecular xenomonitoring; PNG, Papua New Guinea.

Microfilaria prevalence was significantly associated with MX rate (*R*^2^ = 0.49, *P* < .001). The inclusion of primary vector genus as a covariate did not improve the predictive power of the model. The strength and slope of the relationship between MX rate and mf prevalence were lowest in study areas that had not yet received MDA and increased in areas that had made greater progress towards elimination (Figure 4A). Within each of these contexts, a large proportion of the variation between measurements of MX remained unexplained. However, data from longitudinal studies showed a strong linear relationship between MX rate and mf prevalence (*R*^2^ = 0.78, *P* < .001) (Figure 4B). Similar declines in both MX rate and mf prevalence were observed in the 4 included study areas over time (Supplementary Figure 2).

## Discussion

The GPELF recommends the implementation of MX alongside TAS for post-MDA surveillance, advising that 5000–10 000 mosquitoes should be screened due to the low expected infection rate in elimination settings [15]. Molecular xenomonitoring has increasingly been taken up by national LF programs [10, 46, 47]. However, utility is limited without an understanding of the relationship between MX rates and disease prevalence. To match the evolving needs of LF programs approaching elimination, the scope of this review was to evaluate the suitability of MX against a range of programmatic goals.

Our primary analysis shows that MX is highly sensitive for detecting filariasis presence compared with mf screening. Even when mf prevalence is low, 100% sensitivity was observed with mosquito sample sizes of 1000 or more. When human prevalence is very low (<0.25%), samples of 4000–6000 mosquitoes achieved 100% sensitivity. With the ability to process mosquitoes in pools, this corresponds to approximately 200 PCR reactions. Of the 2 studies with less than 100% sensitivity, one conducted its mf and MX surveys in somewhat different locations [39] and both featured lag times of 12–18 months between MX and mf surveys. Coupled with very low mf prevalence measurements in each site (<0.3%) and the focal nature of filarial infections after MDA, these factors may explain the reduced sensitivity observed in these studies.

Our secondary analysis shows a significant relationship between MX rates and mf prevalence. The strength of this relationship was higher in areas currently undergoing or having completed MDA compared with pre-MDA settings. In each setting, a large proportion of the variation in MX rate remains unexplained. However, analysis of a limited number of longitudinal studies revealed strong correlations between MX and mf measurements. In these 4 study communities, the MX rate tracked the observed declines in mf prevalence throughout MDA. This finding lends strong support for longer-term monitoring using MX to track progress towards elimination.

The findings of this study should be interpreted with caution due to the limited number of MX studies that reported the corresponding mf prevalence and therefore meeting the inclusion criteria for this review. Few of the included studies primarily aimed to evaluate the accuracy of MX, and consequently the number of paired MX rate and mf prevalence observations for each study is low. As a result, the CIs for our estimates of sensitivity are extremely wide. Further evidence will be required to confirm the sensitivity of MX with a high degree of certainty, particularly in areas of very low mf prevalence. The lack of consistent methodology between the studies must also be acknowledged. Besides the variables that were controlled for in our analyses (MDA status and primary vector), the included studies also employed a variety of mosquito-collection methods. As these demonstrate biases towards different physiological states, MX rates measured with different strategies may differ even when the mf prevalence is constant [48]. Similarly, the different methods used for counting mf may result in different estimates of the human prevalence [49]. Included studies also differed methodologically in terms of the size of area covered, level of sampling intensity, and molecular methods used for parasite detection, as well as environmentally in terms of season, presence of vector control, and vector age structure. While MX sensitivity appeared to be reliably strong despite the inconsistencies, these factors may contribute to the unexplained variation in MX rate at different levels of mf prevalence.

### Conclusions

For MX to have applicability to current LF program thresholds, it must accurately predict mf prevalence values below 1%, the threshold for stopping MDA. The strength of the relationship between the 2 variables provides reason for optimism that MX methods could be used to estimate infection rates. However, the degree of unexplained variation suggests that further work is needed to understand the variables that influence MX rates before they can be used for decisions to stop MDA. This variation appears to be driven, in part, by methodological inconsistencies, and the explanatory power of MX would therefore be strengthened with clear normative guidance for its implementation, including collection methods, frequency, geographical scale, and sample size. Furthermore, MX shows clear potential to detect positive communities where case surveillance does not, and we have shown that presence/absence detection can be achieved with relatively low mosquito sample sizes. Molecular xenomonitoring could therefore play a future role in sensitively detecting resurgence in a noninvasive, sustainable way.

## Supplementary Data

Supplementary materials are available at *Clinical Infectious Diseases* online. Consisting of data provided by the authors to benefit the reader, the posted materials are not copyedited and are the sole responsibility of the authors, so questions or comments should be addressed to the corresponding author.

ciab197_suppl_Supplementary-Material

## Notes

***Acknowledgments.*** We thank Marty Richardson and Peter Diggle for providing guidance on the statistical analyses conducted in this review. We also thank Edward Thomsen for insightful comments in reviewing the manuscript.

***Disclaimer.*** The views, opinions, assumptions, or any other information set out in this article are solely those of the authors and should not be attributed to the funders or any other person connected with the funders.

***Supplement sponsorship.*** This article appears as part of the supplement “Sustainable control of neglected tropical diseases – surveillance and diagnostics”, sponsored by the NTD Modelling Consortium.

***Financial support.*** J. P. was supported by the Medical Research Council (grant number N013514/). L. J. R. received funding from the NTD Modelling Consortium by the Bill and Melinda Gates Foundation (grant number OPP1184344).

***Potential conflicts of interest.*** Both authors: No reported conflicts of interest. Both authors have submitted the ICMJE Form for Disclosure of Potential Conflicts of Interest. Conflicts that the editors consider relevant to the content of the manuscript have been disclosed.
