# Supplementary material for: Evaluating the Diagnostic Test Accuracy of Molecular Xenomonitoring Methods for Characterizing Community Burden of Lymphatic Filariasis
Source: Clin Infect Dis. 2021 Jun 14;72(Suppl 3):S203–9. doi: 10.1093/cid/ciab197 (PMC8201559; doi:10.1093/cid/ciab197)
Supplement: ciab197_suppl_Supplementary-Material [file ciab197_suppl_supplementary-material.docx]

**Supplementary Table 1. Search terms and search strategy used in retrieving the articles.**[NB. In addition to this review, the search strategy has been used for another research project with different inclusion criteria. The aim of this strategy was to identify studies that have utilised molecular xenomonitoring to screen mosquitoes for any filarial worm diseases, including those not transmitted by mosquitoes. The terms *Onchocerca*, *Mansonella* and *Loa* are therefore included in the search terms. However, the specific inclusion criteria for this review ensure that only studies relating to lymphatic filariasis were included.]

| **Search steps** | **Search terminology** |
| --- | --- |
| #1 | Xenosurveillance OR Xeno-surveillance |
| #2 | Xenomonitor* OR Xeno-monitor* |
| #3 | Mosquito* OR Aedes OR Anopheles OR Culex OR Mansonia |
| #4 | “Molecular screen*” OR “Molecular diagnos*” OR PCR OR “Polymerase chain reaction” OR sequencing OR LAMP |
| #5 | #3 AND #4 |
| #6 | #1 OR #2 OR #5 |
| #7 | Onchocerc* OR “River blindness” |
| #8 | Filaria* OR Elephantiasis OR “Wucheria bancrofti” OR “W. bancrofti” OR “Brugia malayi” OR “B. malayi” OR “Brugia timori” OR “B. timori” |
| #9 | Loa OR Loiasis OR “African eye worm” |
| #10 | Mansonel* |
| #11 | #7 OR #8 OR #9 OR #10 |
| #12 | #11 AND #6 |

**Supplementary Table 2: Assessment criteria and marking strategy for evaluating methodological quality**

| **Domain** | **Description** |
| --- | --- |
| Blinding of Index Test | *Were the index test results interpreted without knowledge of the results of the reference standard?*  This item is similar to “blinding” in intervention studies. Interpretation of index test results may be influenced by knowledge of the reference standard. The potential for bias is related to the subjectivity of index test interpretation and the order of testing.   If the index test was always conducted and interpreted prior to the reference standard, this item was rated “low risk”. If it was not described whether the outcomes of the reference standard were known to those conducting the index test, the study was typically considered to have an “unclear risk”. However, if the objective of the study was not to evaluate the use of the index test, such cases were also considered to be “low risk”. |
| Blinding of Reference Test | *Were the reference standard results interpreted without knowledge of the results of the index test?*  This item is similar to the signalling question related to interpretation of the index test. Potential for bias is related to the potential influence of prior knowledge on the interpretation of the reference standard.   As above, if it was not described whether the outcomes of the index test were known to those conducting the reference standard, the study was typically considered to have an “unclear risk”. However, if the objective of the study was not to evaluate the use of the index test, such cases were also considered to be “low risk”. |
| Length of Time Between Surveys | *Was the interval between index test and reference standard appropriate?*   Ideally, index test and reference standard surveys would be collected in the same communities at the same time. If there is a significant delay, or if MDA has been implemented between the index test and reference standard, misclassification may occur due to an increase or decrease in disease prevalence between the two surveys.  We considered a gap of less than 6 months between to be “low” risk. For gaps of more than 6 months and less than 12 months, or if the length of time between surveys was not described, we considered the risk to be “unclear”. For gaps of more than 12 months but less than 18 months the risk was considered “high”. If the gap was more than 18 months, or if MDA had been implemented in between, the comparison was considered unsuitable for inclusion in the review. |
| Matching of Sampled Communities | *Were the communities in which the index test and reference standards were conducted appropriately matched?*  Ideally, the index test and reference standard surveys would be conducted in exactly the same areas. If the surveyed area is large, eg. district level, with MX surveys conducted in one village and mf surveys conducted in a distant village, the surveys may not be expected to provide comparable results.   We considered surveys that were undertaken in the same villages to be “low” risk, even if the households targeted for entomological and parasitological surveys were not identical. However, if surveys were conducted at a district or sub-district level and MX surveys were carried out in specific communities within the district or sub-district that was not matched by mf surveys, we considered this to be “high” risk. If there was not information about sampling strategies to form a judgement, studies were graded as “unclear” risk |
| Continuity of Methods  [Longitudinal studies only] | *Could variation in methodology used at different time points have introduced bias?*  Any variation in the methodology used for the sampling of locations, the index test or reference standard may lead to systematic changes in the outcome data collected.   We considered studies to be at “low” risk if there was no or limited variation in methodology, and “high” risk if there was significant variation that we felt could influence measurements of MX rate and mf prevalence. If there was not information about sampling strategies to form a judgement, studies were graded as “unclear” risk. |
| Index test participant applicability | *Do the index test participants match the review question?*  We considered studies to be at “high” risk if we had concerns that the sampling strategy targeted a limited population that was not widely applicable to the general mosquito population in the area. We considered studies to be at “low” risk if we had no such concerns, and “unclear” if there was not enough information to make a decision. |
| Reference test participant applicability | *Do the reference test participants match the review question?*  We considered studies to be at “high” risk if we had concerns that the sampling strategy targeted a limited population that was not widely applicable to the general human population in the area. We considered studies to be at “low” risk if we had no such concerns, and “unclear” if there was not enough information to make a decision. |

**Supplementary Dataset 1. Extracted Data from Included Studies**

All data extracted from included studies has been made available in an online database (Open Science Framework: DOI 10.17605/OSF.IO/MX2UZ)


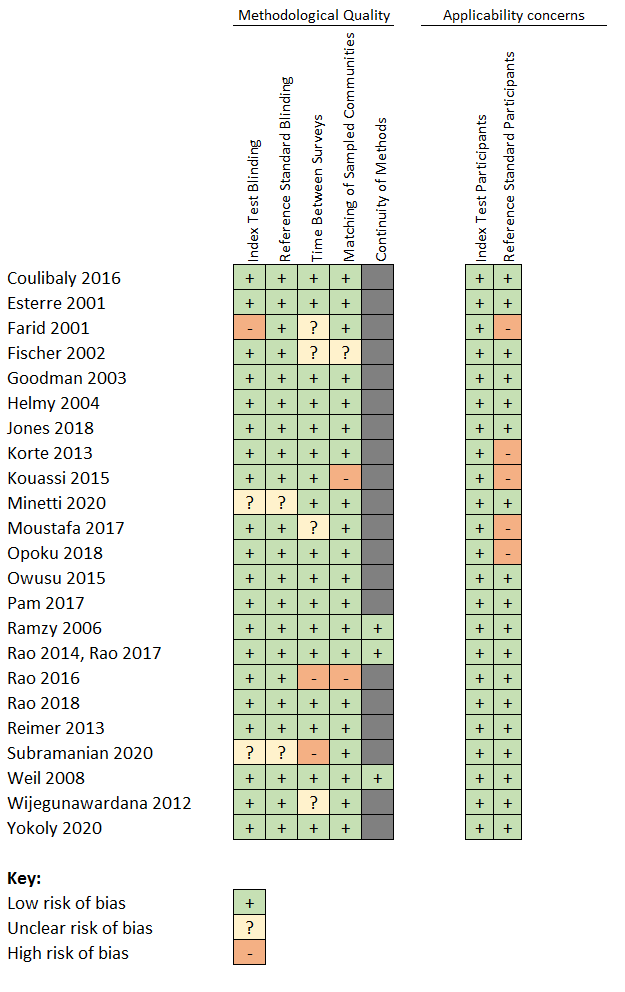


**Supplementary Figure 1. Summary of the results of the methodological quality assessment for each study. Continuity of methods was only assessed for studies that conducted mf and MX surveys at three or more timepoints.**

**Supplementary Table 3. Explanations for assessments of methodological quality**

|  | Risk of Bias | | | | |  | Applicability domain | |
| --- | --- | --- | --- | --- | --- | --- | --- | --- |
|  | Index Test blinded | Reference standard blinded | Appropriate gap between surveys | Appropriate matching of sampled populations | Continuity of methodology over time |  | Applicability of participants for index | Applicability of participants for reference |
| **Coulibaly 2016** | Low - Not described but unlikely to have caused a bias | Low - Not described but unlikely to have caused a bias | Low - Gap between parasitological and entomological surveys was less than 6 months | Low - Entomological and parasitological surveys were conducted in the same six villages | N/A |  | Low - No concerns | Low - No concerns |
| **Esterre 2001** | Low - Not described but unlikely to have caused a bias | Low - Not described but unlikely to have caused a bias | Low - Gap between parasitological and entomological surveys was less than 6 months | Low - Mosquitoes and humans were sampled from all inhabited areas | N/A |  | Low - No concerns | Low - No concerns |
| **Farid 2001** | High - Probable that the mf prevalence in each village was known prior to the study being conducted, and the objectives of the study could have provided a motivation for the diagnostic test to reflect a higher prevalence in the high mf district | Low - Probable that the entomological assessments were made after the reference standard had been measured. | Unclear - Gap between parasitological and entomological surveys not described | Low - Each village was divided into nine blocks for sampling to ensure coverage of the entire village from which humans were enrolled | N/A |  | Low - No concerns | High - sample population limited to households containing children in one specific year group |
| **Fischer 2002** | Low - Not described but unlikely to have caused a bias | Low - Not described but unlikely to have caused a bias | Unclear - Gap between parasitological and entomological surveys not described | Unclear - Mosquito sampling strategy not described | N/A |  | Low - No concerns | Low - No concerns |
| **Goodman 2003** | Low - Not described but unlikely to have caused a bias | Low - Not described but unlikely to have caused a bias | Low - Gap between parasitological and entomological surveys was less than 6 months | Low - 4 sentinel sites in the commune provided matching entomological and parasitological data | N/A |  | Low - No concerns | Low - No concerns |
| **Helmy 2004** | Low - Not described but unlikely to have caused a bias | Low - Not described but unlikely to have caused a bias | Low - Gap between parasitological and entomological surveys was less than 6 months | Low - Sampling strategy was similar for both survey types | N/A |  | Low - No concerns | Low - No concerns |
| **Jones 2018** | Low - Not described but unlikely to have caused a bias | Low - Not described but unlikely to have caused a bias | Low - Gap between parasitological and entomological surveys was less than 6 months | Low - Entomological and parasitological surveys were conducted in the same villages | N/A |  | Low - No concerns | Low - No concerns |
| **Korte 2013** | Low - Not described but unlikely to have caused a bias | Low - Not described but unlikely to have caused a bias | Low - Exact dates not described but both surveys were indicated as being conducted in 2008 -2009, with no MDA conducted in the region at any stage | Low - Entomological and parasitological surveys were performed in the same neighbourhoods. | N/A |  | Low - No concerns | High - Surveys were limited to those enrolled in night school and a small number in un-sanitised communities, these may not capture a representative sample of the population |
| **Kouassi 2015** | Low - Not described but unlikely to have caused a bias | Low - Not described but unlikely to have caused a bias | Low - Gap between parasitological and entomological surveys was less than 6 months | High - In each of the five districts, parasitological surveys recruited people at random from across the district. Entomological surveys were focused in 15 specific sub-sectors selected based on potential exposure to mosquito bites | N/A |  | Low - No concerns | High - exclusively used volunteers aged 15 and over, may not be representative of the prevalence in the overall population |
| **Minetti 2020** | Unclear - Not described whether those processing the index test results were aware of the mf prevalence for that community, and the objectives of the study could have provided a motivation for the diagnostic test to reflect a higher prevalence in the high mf district | Unclear - Not described whether those processing the reference test results were aware of the index test results for that community, and the objectives of the study could have provided a motivation for the diagnostic test to reflect a higher prevalence in the high mf district | Low - Gap between parasitological and entomological surveys was less than 6 months | Low - Entomological and parasitological surveys were conducted in the same villages | N/A |  | Low - No concerns | Low - No concerns |
| **Moustafa 2017** | Low - Not described but unlikely to have caused a bias | Low - Not described but unlikely to have caused a bias | Unclear - 6 month gap in between parasitological and entomological surveys | Low - Entomological and parasitological surveys were conducted in the same villages | N/A |  | Low - No concerns | High - children aged 6-7 only |
| **Opoku 2018** | Low - Not described but unlikely to have caused a bias | Low - Not described but unlikely to have caused a bias | Low - In the Northern region, parasitological and entomological surveys were conducted in the same month. In the Western region, the gap was approximately 11 months. Overall the risk of bias resulting from timing of this study was considered to be low. | Low - Entomological and parasitological surveys were conducted in the same villages | N/A |  | Low - No concerns | High - exclusively used volunteers aged 16 and over, may not be representative of the prevalence in the overall population |
| **Owusu 2015** | Low - Not described but unlikely to have caused a bias | Low - Not described but unlikely to have caused a bias | Low - Gap between parasitological and entomological surveys was less than 6 months | Unclear - Parasitological survey sampling strategy is not described. | N/A |  | Low - No concerns | Low - No concerns |
| **Pam 2017** | Low - Not described whether those processing the index test results were aware of the mf prevalence for that community, though given the objectives of the study this is unlikely to have caused a bias | Low - Not described whether those processing the reference test results were aware of the index test results for that community, though given the objectives of the study this is unlikely to have caused a bias. | Low - Gap between parasitological and entomological surveys was less than 6 months | Low - Entomological and parasitological surveys were conducted in the same communities | N/A |  | Low - No concerns | Low - No concerns |
| **Ramzy 2006** | Low - Not described but unlikely to have caused a bias | Low - Not described but unlikely to have caused a bias | Low - Gaps between parasitological and entomological surveys were less than 6 months | Low - Sampling strategy was similar for both survey types | Low - No concerns about continuity of methodology |  | Low - No concerns | Low - No concerns |
| **Rao 2014, Rao 2017** | Low - Not described but unlikely to have caused a bias | Low - Not described but unlikely to have caused a bias | Low - Exact timing of entomological surveys not described, but the year conducted matches the year in which parasitological surveys were conducted for each area. | Low - In each of the 19 Public Health Inspector (PHI) study areas, a systematic sampling strategy was used for both survey types, ensuring coverage of all areas of each of the sub-level Public Health Midwife (PHM) areas in the PHI. | Low - No concerns about continuity of methodology |  | Low - No concerns | Low - No concerns |
| **Rao 2016** | Low - Not described but unlikely to have caused a bias | Low - Not described but unlikely to have caused a bias | High - approximately 1 year between parasitological and entomological surveys | High - In each of the 19 Ministry of Health (MOOH) study areas, the sampling strategy for parasitological surveys included participants from every sub-level Public Health Midwife (PHM) area (340 PHMs in total). In contrast, only 60 PHMs were sampled for the entomological surveys. | N/A |  | Low - No concerns | Low - No concerns |
| **Rao 2018** | Low - Not described but unlikely to have caused a bias | Low - Not described but unlikely to have caused a bias | Low - Gaps between parasitological and entomological surveys were less than 6 months | Low - Sampling strategy was similar for both survey types | N/A |  | Low - No concerns | Low - No concerns |
| **Reimer 2013** | Low - Not described but unlikely to have caused a bias | Low - Not described but unlikely to have caused a bias | Low - Gaps between parasitological and entomological surveys were less than 6 months | Low - Entomological and parasitological surveys were conducted in the same villages | N/A |  | Low - No concerns | Low - No concerns |
| **Subramanian 2020** | Unclear - Not described whether those processing the index test results were aware of the mf prevalence for that community | Unclear - Not described whether those processing the reference test results were aware of the index test results for that community | High - >12 month gap between parasitological and entomological surveys | Low - Entomological and parasitological surveys were conducted in the same villages. 5 households were targeted for mosquito collections and an average of 75 households were targeted for microfilaremia surveys. | N/A |  | Low - No concerns | Low - No concerns |
| **Weil 2008** | Low - Not described but unlikely to have caused a bias | Low - Not described but unlikely to have caused a bias | Low - Gaps between parasitological and entomological surveys were less than 6 months | Low - Entomological and parasitological surveys were conducted in the same villages | Low - No concerns about continuity of methodology |  | Low - No concerns | Low - No concerns |
| **Wijegunawardana 2012** | Low - Not described but unlikely to have caused a bias | Low - Not described but unlikely to have caused a bias | Unclear - Gap between parasitological and entomological surveys not described | Low - Entomological and parasitological surveys were conducted in 2 matching sentinel sites within each district | N/A |  | Low - No concerns | Low - No concerns |
| **Yokoly 2020** | Low - Not described but unlikely to have caused a bias | Low - Not described but unlikely to have caused a bias | Low - Gaps between parasitological and entomological surveys were less than 6 months | Low - Entomological and parasitological surveys were conducted in 2 matching sentinel sites within each district | N/A |  | Low - No concerns | Low - No concerns |


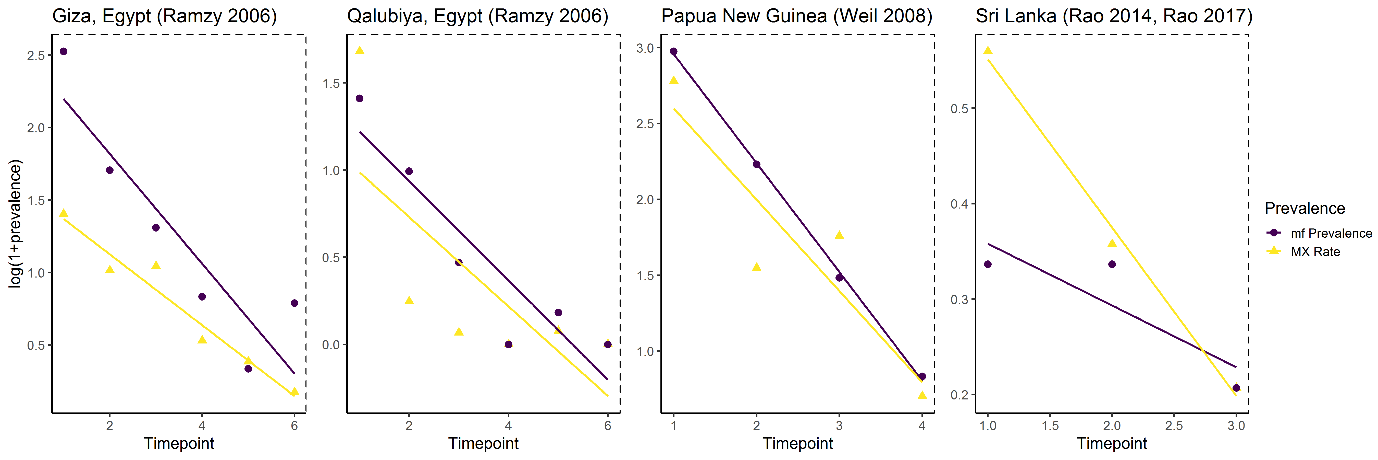


**Supplementary Figure 2. Observations of mf prevalence and MX rate over time. Longitudinal data were provided from three studies (Ramzy *et al.,* 2006, Weil *et al.,* 2008, and Rao *et al.,* 2014/Rao *et al.,* 2017).**
